# Transcranial magnetic stimulation for catatonia: case series

**DOI:** 10.1192/j.eurpsy.2023.2164

**Published:** 2023-07-19

**Authors:** G. Mamedova, N. Zakharova

**Affiliations:** Mental-health Clinic No. 1 named after N.A. Alexeev, Moscow Healthcare Department, Moscow, Russian Federation

## Abstract

**Introduction:**

Catatonia is diagnosed in 5–43% of patients with various mental disorders, thus actualizing the problem of elaborating therapeutic interventions for catatonia on an outpatient basis. Although the current experience in application of transcranial magnetic stimulation (TMS) in catatonia is limited, it provides promising data on positive effect of dorsolateral prefrontal cortex (DLPFC) stimulation in a series of clinical observations. According to the available data, TMS shows comparable efficacy with electroconvulsive therapy, but unlike it is safe and does not require general anesthesia in intensive care unit.

**Objectives:**

to evaluate the efficacy and safety of TMS in the treatment of catatonia in patients with mental disorders

**Methods:**

Four patients were diagnosed with catatonia as part of schizophrenia spectrum disorders in three cases (P1,4,7) and in one case within the structure of recurrent depression phase (P8). Psychopathological examination includes PANSS, SAS, NSA-4, BFCRS, NCRS, and BACS.

Personalized choice of stimulation protocol was determined by rCBF lateralization in DLPFC reflecting the neuronal activity in that region: 1) P1, P4, and P8 underwent 20 sessions of high-frequency stimulation at the frequency of 20 Hz with the amplitude of 120% MT in the projection of left DLPFC 2) P7 underwent 20 sessions of low-frequency stimulation at the frequency of 1 Hz with the amplitude of 120% MT in the projection of right DLPFC

**Results:**

Safety evaluation was performed daily during TMS sessions. None of participants reported any adverse events at high compliance.

The efficacy was estimated during by the following criteria: 1) positive clinical response: decline of BFCRS and NCRS scores by 70% from the primary evaluation 2) achievement symptomatic remission (total BFCRS and NCRS score 3 and less).

Positive clinical response was detected in all four patients, however, symptomatic remission was formed only in two of them (P1 and P4) referring to BFCRS.

Evaluation of neurotransmitter concentration: P1, P7, P8 showed a tendency for absolute and relative glutamate concentration values to approach normal. After the TMS course GABA concentration diminished in all cases but P4, in whom the elevation of GABA level was registered.

**Conclusions:**

TMS potentially activates metabolic processes in brain tissues, thus promoting deceleration of pathological mechanisms and potentiating neuroplasticity with procognitive effect, expressed primarily in the increase of processing speed and response to it, as well as in the improvement of working memory. To summarize, the influence of TMS on local brain regions makes it possible to achieve a positive clinical effect in treatment of catatonia.

No strong and unequivocal results were received for the efficacy of TMS in treatment of catatonia. A positive clinical effect was seen, however, insufficient for achieving remission in the majority of subjects.

**Disclosure of Interest:**

None Declared

